# Specificity Determinants of the Silkworm Moth Sex Pheromone

**DOI:** 10.1371/journal.pone.0044190

**Published:** 2012-09-05

**Authors:** Pingxi Xu, Antony M. Hooper, John A. Pickett, Walter S. Leal

**Affiliations:** 1 Honorary Maeda-Duffey Laboratory, University of California Davis, Davis, California, United States of America; 2 Rothamsted Research, West Common, Harpenden, Hertfordshire, United Kingdom; Plant and Food Research, New Zealand

## Abstract

The insect olfactory system, particularly the peripheral sensory system for sex pheromone reception in male moths, is highly selective, but specificity determinants at the receptor level are hitherto unknown. Using the *Xenopus* oocyte recording system, we conducted a thorough structure-activity relationship study with the sex pheromone receptor of the silkworm moth, *Bombyx mori,* BmorOR1. When co-expressed with the obligatory odorant receptor co-receptor (BmorOrco), BmorOR1 responded in a dose-dependent fashion to both bombykol and its related aldehyde, bombykal, but the threshold of the latter was about one order of magnitude higher. Solubilizing these ligands with a pheromone-binding protein (BmorPBP1) did not enhance selectivity. By contrast, both ligands were trapped by BmorPBP1 leading to dramatically reduced responses. The silkworm moth pheromone receptor was highly selective towards the stereochemistry of the conjugated diene, with robust response to the natural (10*E,*12*Z*)-isomer and very little or no response to the other three isomers. Shifting the conjugated diene towards the functional group or elongating the carbon chain rendered these molecules completely inactive. In contrast, an analogue shortened by two omega carbons elicited the same or slightly higher responses than bombykol. Flexibility of the saturated C1–C9 moiety is important for function as addition of a double or triple bond in position 4 led to reduced responses. The ligand is hypothesized to be accommodated by a large hydrophobic cavity within the helical bundle of transmembrane domains.

## Introduction

The identification of bombykol, (10*E*,12*Z*)-hexadecadien-1-ol (**1**), the sex pheromone for the silkworm moth, *Bombyx mori* [1], more than five decades ago triggered physiologists’ interest in insect olfaction, and paved the way for current molecular studies. Probing the system with earlier techniques such as electroantennogram (EAG) and single-sensillum recordings (SSR), pioneers in the field unraveled an inordinate sensitivity and selectivity of the insect’s olfactory system [2]. These earlier studies clearly demonstrated that structural modifications dramatically reduce neuronal responses or render the molecules completely inactive [3], but it remains mostly unknown how pheromone molecules interact with odorant receptors (ORs) housed in these neurons, although various moth sex pheromone receptors have been de-orphanized to date [4]–[11]. To identify pheromone specificity determinants, we challenged with a panel of bombykol analogs the silkworm moth sex pheromone receptor, BmorOR1, co-expressed with its obligatory co-receptor, BmorOrco [4] in the *Xenopus* oocyte system. As the BmorOR1•BmorOrco-expressing oocytes showed robust and moderate responses to bombykol and bombykal, respectively, we investigated whether a functional recombinant pheromone-binding protein, BmorPBP1 [12], would enhance selectivity. Here, we provide strong evidence that bombykol does not require BmorPBP1 to activate BmorOR1. Additionally, we show that the stereochemistry of the double bonds, flexibility of saturated moiety, the functional group, and the number of carbons atoms after the unsaturations are specificity determinants of the pheromone molecule.

## Results and Discussion

### Selectivity of the Functional Group

**Figure 1:**
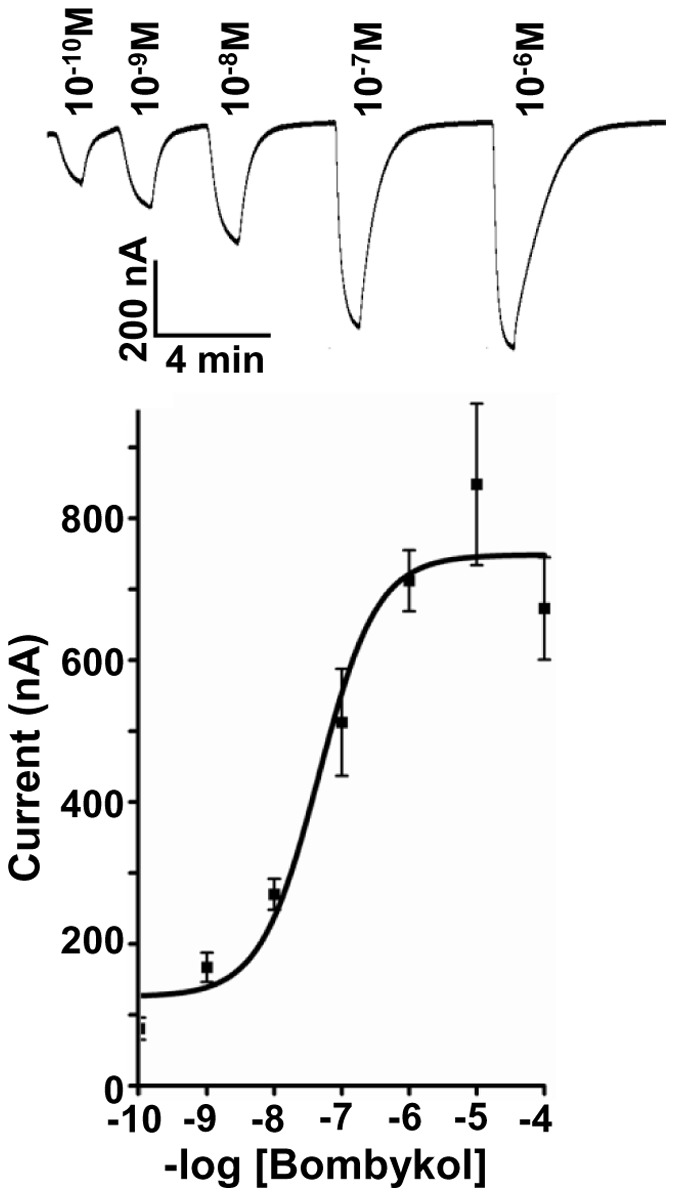
Bombykol receptor expressed in the *Xenopus* oocyte recording system. Robust currents from BmorOR1•BmorOrco-expressing oocytes when perfused with bombykol, and dose-dependent responses. *n* = 3–5, error bars in all figures represent SEM.

**Figure 2:**
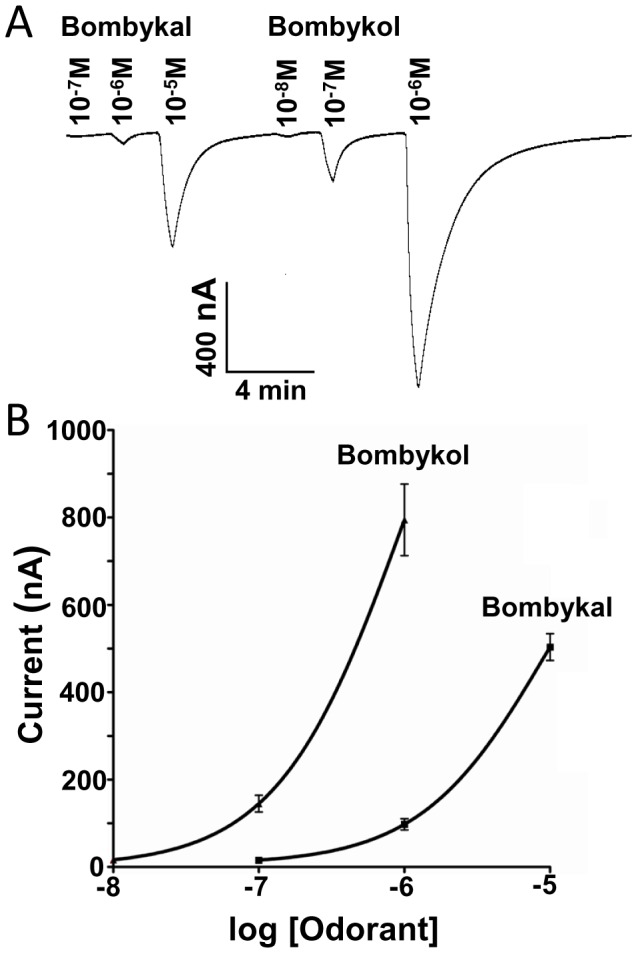
Activation by bombykal. (*A*) Current responses and (*B*) dose-dependent relationships obtained by challenging BmorOR1•BmorOrco-expressing oocytes with increasing concentrations of bombykol and bombykal. *n* = 6.

**Figure 3:**
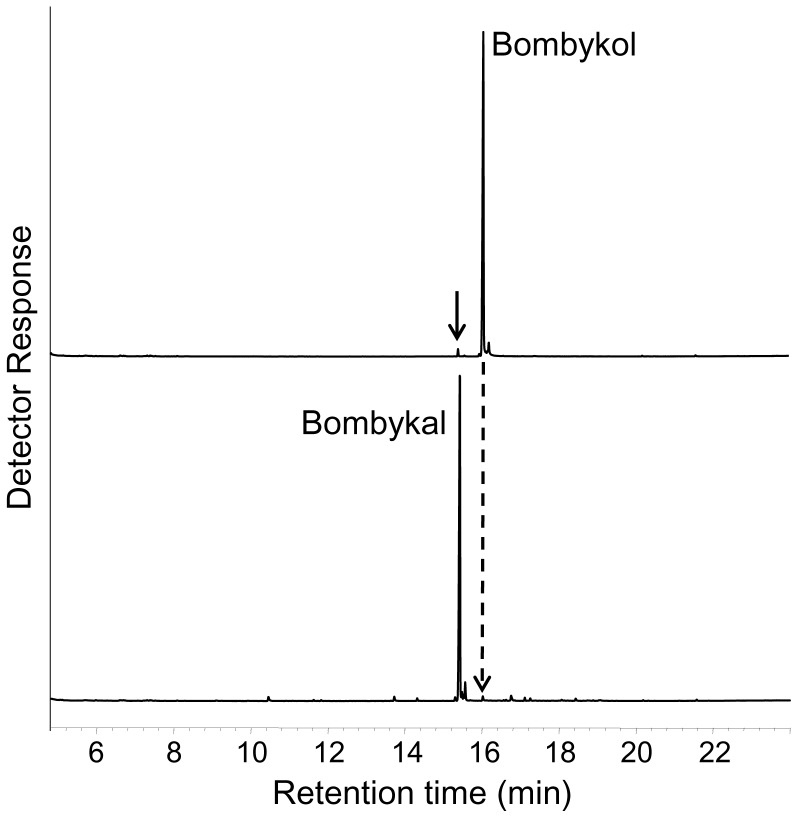
Chemical analysis of synthetic pheromone components. GC-MS traces obtained from bombykol (upper trace) and bombykal (lower trace) samples freshly prepared to challenge BmorOR1•BmorOrco-expressing oocytes. Arrow indicates trace amounts of bombykal (1.3%) in the bombykol sample, whereas a dotted arrow shows traces of bombykol (0.9%) in bombykal sample. The ratio of bombykol (retention time, 16.06 min) to bombykal (15.42 min) in the two samples was 1.015±0.02, *n* = 3.

First, we examined the response of BmorOR1•BmorOrco-expressing oocytes to bombykol. The silkworm moth receptor responded to the sex pheromone in a dose-dependent fashion (EC_50_ 4.54×10^−8 ^M) and with a remarkable low threshold (<0.1 nM) (Figure 1). Then, we compared the OR responses elicited by bombykol and bombykal. The literature is dichotomous regarding the selectivity of BmorOR1 towards these two components of the silkworm moth’s sex pheromone system [3]. Using the *Xenopus* oocyte recording system, it has been shown that BmorOR1•BmorOrco is narrowly tuned to bombykol [4]. By contrast, it has been reported that BmorOR1-expressing HEK 293 cells responded almost equally to bombykol and bombykal [5]. In our hands, BmorOR1•BmorOrco-expressing oocytes were indeed more sensitive to bombykol, but responded to bombykal with about one order of magnitude higher threshold (Figure 2). After activation stimulus was applied, oocytes were thoroughly washed until a steady baseline was reached. To save odorant samples and expedite these recovery times, all comparative studies were made by injecting test odorants rather than by perfusion, and comparative EC_50_s were calculated on the basis of source doses. Therefore, they are underestimation of the actual EC_50_s. The comparative EC_50_ for bombykol and bombykal were 9.9×10^−7^M and 9.6×10^−6^M, respectively (Figure 2). We analyzed our synthetic samples just prior to electrophysiological recordings to avoid possible misinterpretation derived from sample quality. There are two potential problems to consider, i.e., aldehydes are prone to degradation through auto-oxidation leading to lower than nominal concentrations and the bombykal sample may contain considerable amounts of unreacted bombykol (used as starting material). Our chemical analysis indicated that the two samples had the same concentration and that bombykol contamination in bombykal samples is very low (<0.9%) (Figure 3). If the response would be elicited by residues of bombykol in the bombykal samples, one would expect at least 2 orders of magnitude differences. Interestingly, the responses of the “naked receptor” differ from the neuronal activity of the olfactory system of the silkworm, which showed no cross-over whatsoever, with the bombykol and bombykal neurons responding specifically to the alcohol and aldehyde, respectively [2], [3]. It has been suggested that addition of a pheromone-binding protein, BmorPBP1, to the HEK 293 cell system restores selectivity [5].

### Bombykol and Bombykal are “Trapped” by BmorPBP1

**Figure 4:**
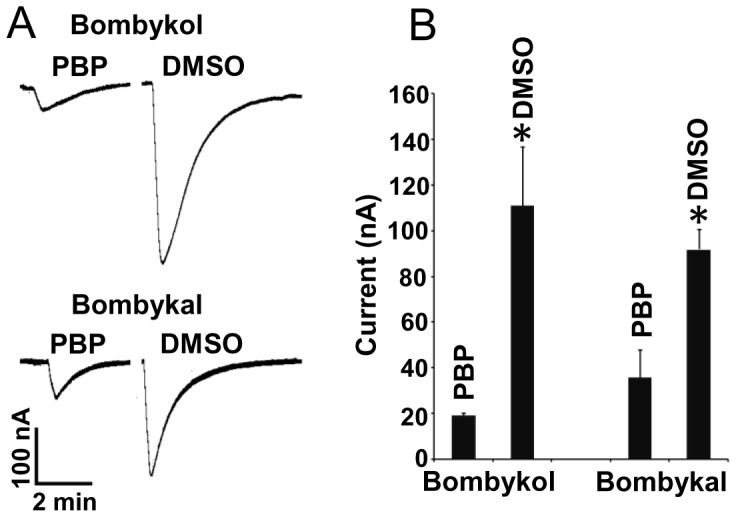
Synthetic pheromone components trapped by PBP. (*A*) Traces and (*B*) quantification of current responses obtained from the BmorOR1•BmorOrco-expressing oocytes when presented with bombykol and bombykal solubilized either by DMSO or BmorPBP1. *n* = 3. *Significantly different (t-test, P<0.05).

In an attempt to reconcile the data in the literature we investigated whether addition of PBP would enhance selectivity of the BmorOR1•BmorOrco receptor complex when expressed in *Xenopus* oocytes. We compared the receptor responses to bombykol and bombykal solubilized either by DMSO or BmorPBP1. Interestingly, receptor activity was dramatically reduced when the ligands were solubilized by BmorPBP1 (Figure 4). Bombykol (1 µM) dissolved in DMSO elicited robust receptor response, but very weak response when solubilized by BmorPBP1. Here, the ratio of BmorPBP1 to bombykol was 10∶1. Bombykal (10 µM) elicited strong response when dissolved in DMSO and weak response when solubilized by BmorPBP1. The receptor response to bombykal solubilized by BmorPBP1 was on average ca. 34% of the response to the same ligand in DMSO, whereas for bombykol the ratio was 13%. This relatively higher response to bombykal in PBP might be merely because of the ratio of PBP:ligand. Given that bombykal requires a 10x higher dose, we prepared samples at a 1∶1 ratio, whereas bombykol samples had a 10∶1 protein/ligand ratio. These findings suggest that in *Xenopus* oocyte there are no negatively-charged surfaces in the vicinity of the receptors or the vitelline membrane surrounding the oocytes prevents the PBP-odorant complexes from interacting with regions of localized low pH, which are necessary to trigger a conformational change that “ejects” ligands from PBP•pheromone complexes [12]–[14]. Regardless, the robust responses recorded without PBPs (Figures 1 and 2) strongly suggest that, unlike what has been demonstrated for Obp76a = LUSH [15], [16] in *D. melanogaster*, PBP-pheromone complexes are not necessary for activation of moth ORs.

### Pheromone Stereochemistry

**Figure 5:**
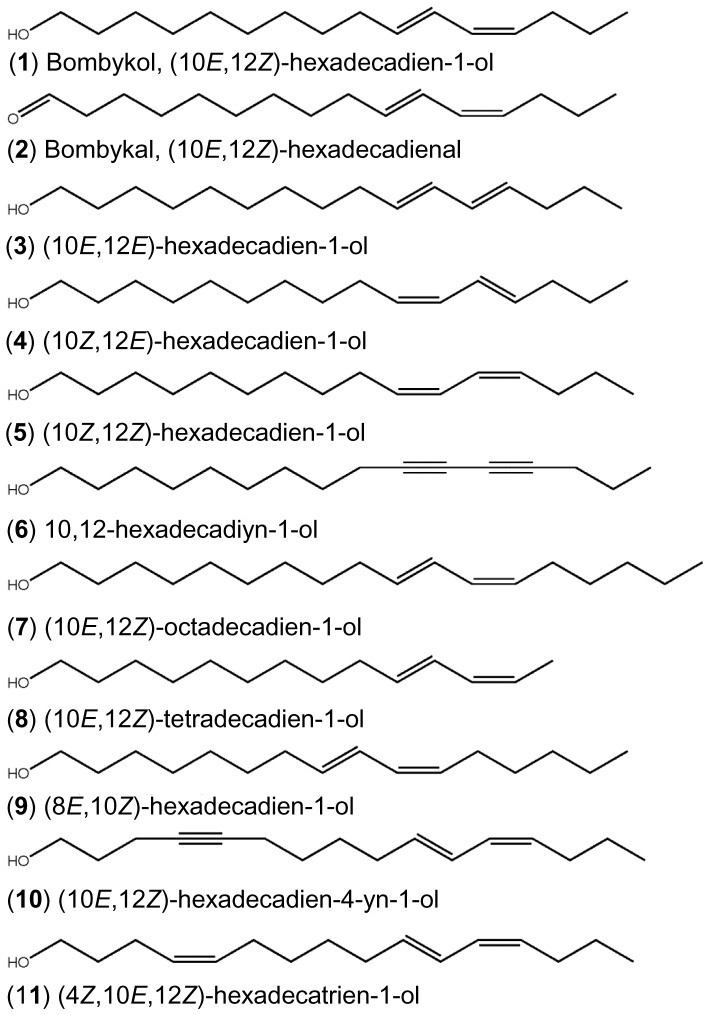
Chemical structures. Structures of the silkworm moth sex pheromone (**1**) and bombykol-related compounds, which were used to challenge BmorOR1•BmorOrco-expressing oocytes.

**Figure 6:**
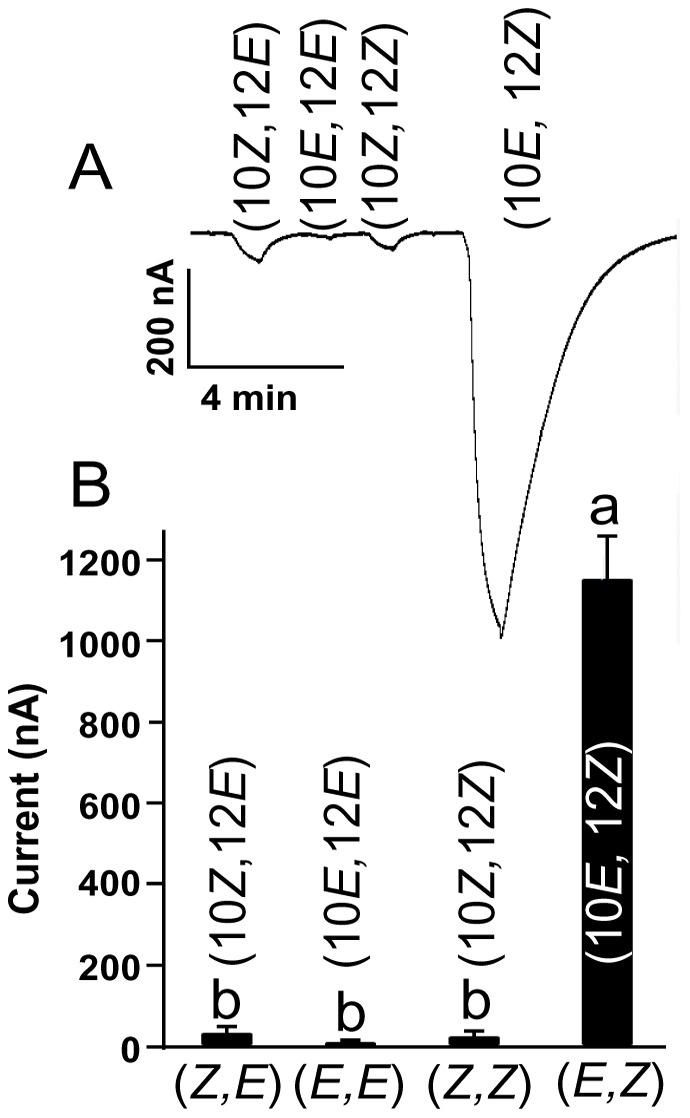
Stereochemical selectivity. (*A*) Traces and (*B*) quantification of current responses from BmorOR1•BmorOrco-expressing oocytes perfused with four isomers of bombykol at 0.1 µM. *n* = 5. Bars with the same letter arwe not significantly different (One-way ANOVA, P<0.01).

**Figure 7:**
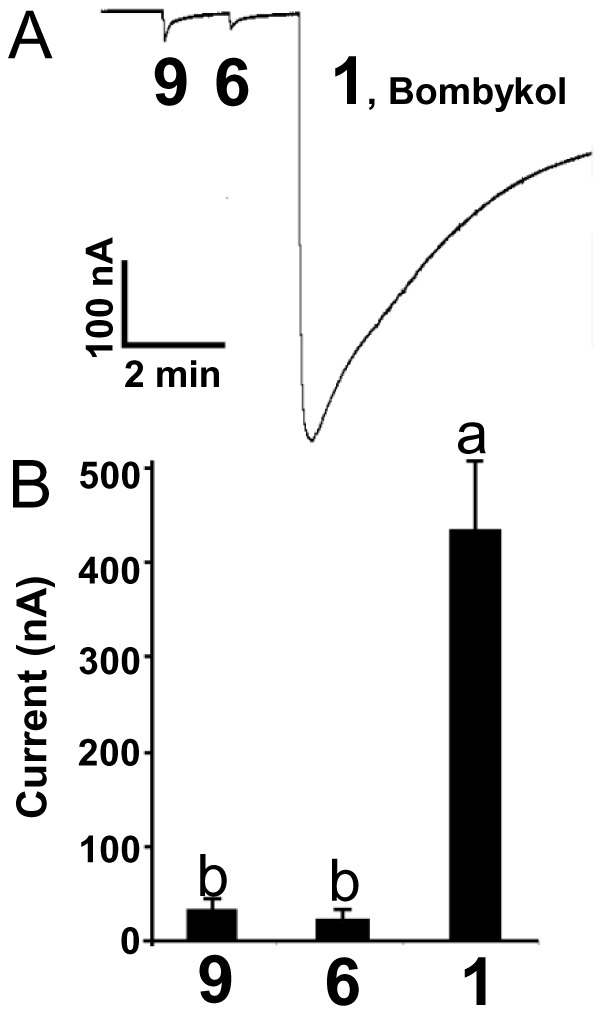
Effect of altering unsaturation on receptor response. (*A*) Traces and (*B*) quantification of current responses elicited by (8*E*,10*Z*)-hexadecadien-1-ol (**9**) and 10,12-hexadecadiyn-1-ol (**6**) presented at 1 mM. *n* = 3. Bars with the same letter are not significantly different (One-way ANOVA, P<0.01).

**Figure 8:**
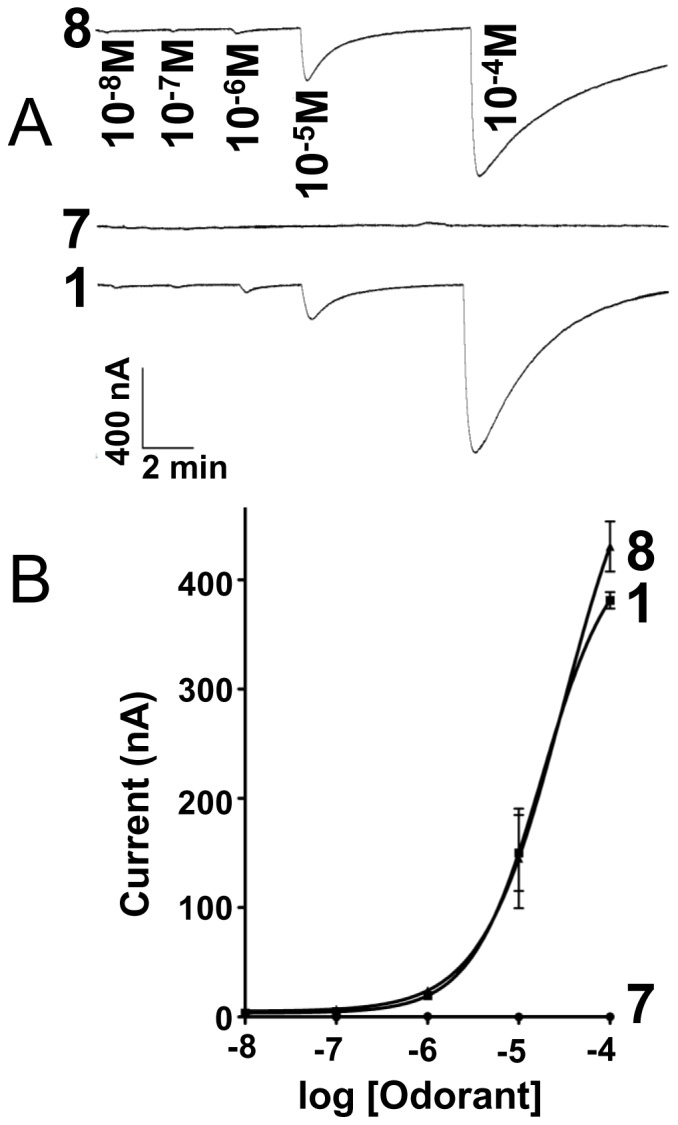
Effect of number of carbons distal to unsaturation. (*A*) Current responses obtained by challenging BmorOR1•BmorOrco-expressing oocytes with bombykol (lower trace, positive control), (10*E*,12*Z*)-octadecadien-1-ol (**7**), and (10*E*,12*Z*)-tetradecadien-1-ol (**8**), robust response at 10 µM. (B) Dose-dependent relationships; *n* = 4.

It is well-known that position and configuration of unsaturation plays a crucial role in pheromone chemistry, but it is unknown if specificity is determined by pheromone receptors alone or in combination with other olfactory proteins. We tested the four possible isomers of bombykol (compounds **1**, **3–5**, Figure 5) and found that BmorOR1•BmorOrco-expressing oocytes respond with high intensity only to the natural stereoisomer of bombykol, (10*E,*12*Z*)-hexadecadien-1-ol, with very low responses to the (10*Z,*12*E*)- and (10*Z,*12*Z*)-isomers, and no response to the (10*E,*12*E*)-isomer (Figure 6). These findings suggest that stereochemistry selectivity is mediated entirely by the receptor. This is in line with the experimental observation that, albeit with different affinities, all four geometric isomers of bombykol bind to the pheromone-binding protein, BmorPBP1 [17]. We also tested whether these double bonds could be replaced by triple bonds, but the receptor was not activated by 10,12-hexadecadiyn-1-ol (**6**) (Figure 7). Next, we compared the effect of the alkyl moiety distal to the unsaturation. Elongating the bombykol molecule by adding two omega carbons renders (10*E*,12*Z*)-octadecadien-1-ol (**7**) completely inactive (Figure 8). However, truncating two omega carbons led to a molecule with apparent higher affinity for the odorant receptor. Indeed, BmorOR1•BmorOrco receptor complex responded to (10*E*,12*Z*)-tetradecadien-1-ol (**8**) with nearly the same or even slightly higher intensity than that elicited by the native ligand, bombykol (Figure 8). Contrary to the stringent requirement for unsaturation with the proper stereochemistry, our findings suggest that the binding pocket in BmorOR1•BmorOrco can accommodate a shorter ligand thus begging questions about the length and flexibility of the moiety between the functional group and unsaturation.

### Flexibility and Length of the C1–C9 Saturated Moiety

**Figure 9:**
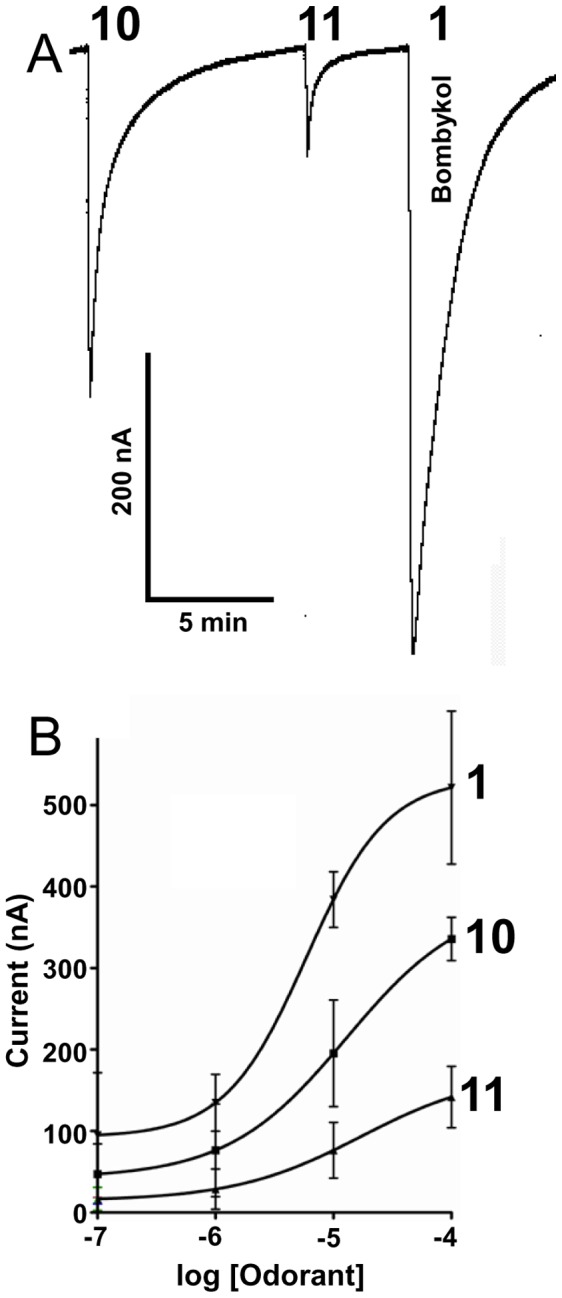
Reducing responses by adding rigidity to the C1–C9 moiety. (*A*) Current responses elicited by (10*E*,12*Z*)-hexadecadien-4-yn-1-ol (**10**), (4*Z*,10*E*,12*Z*)-hexadecatrien-1-ol (**11**), and bombykol (**1**) from BmorOR1•BmorOrco-expressing oocytes (ligands presented at 10 µM). EC_50_s 1.7×10^−5^M, 1.3×10^−5^M, and 5.9×10^−6^M, respectively. (B) Dose-dependent relationships, *n* = 3–4.

To evaluate the positional effect of the unsaturation, we tested another ligand with the double bonds shifted towards the functional group, i.e., (8*E*,10*Z*)-hexadecadien-1-ol (**9**). This ligand showed minimal activation of the BmorOR1•BmorOrco receptor complex (Figure 7) thus implying that the length between the unsaturation and functional group is critical for receptor activation. To determine if the flexibility generated by an unsaturated moiety is important, we tested two bombykol-related compounds each with an additional unsaturation between the functional group and the conjugated double bond moiety. The moderate and low responses elicited by (10*E*,12*Z*)-hexadecadien-4-yn-1-ol (**10**) and (4*Z*,10*E*,12*Z*)-hexadecatrien-1-ol (**11**), respectively (Figure 9), strongly suggest that flexibility of the unsaturated moiety is essential for fitting into the binding pocket, particularly given the stronger effect of the double than the triple bond.

### Conclusions

Structure activity analysis showed that the most important features of the sex pheromone of the silkworm moth are the stereochemistry of a conjugated diene, and the length and flexibility of the hydrocarbon moiety between the diene and the hydroxyl functional group. The length of the hydrocarbon chain distal from the diene moiety is limited to two carbons as in the natural pheromone, but a shorter version elicited as high activity in the receptor as bombykol. BmorOR1•BmorOrco-expressing oocytes responded not only to bombykol, but also to bombykal. Addition of BmorPBP1 did not enhance selectivity, but dramatically reduced current responses thus suggesting that ligands are trapped. The requirements for a large hydrophobic cavity strongly suggest that the yet-to-be-identified binding site in BmorOR1 might be buried in the transmembrane domain.

## Materials and Methods

### Chemicals

Bombykol and bombykal were purchased from Plant Research International (Wageningen, The Netherlands) and kept sealed under helium at −80°C until use. For synthesis, solvents were dried by distillation over CaH_2_ (benzene, dichloromethane) or sodium wire (tetrahydrofuran) or over dry potassium hydroxide (piperidine, pyrrolidine).

### Chemical Analysis

Nuclear magnetic resonance spectroscopy was performed using a Bruker Avance 500 MHz instrument and deuteriochloroform as solvent. Mass spectra were recorded on a Mat95 XP magnetic sector mass spectrometer (Thermo Finnigan). Ionization was by electron impact at 70eV in positive ion mode with a source temperature of 220°C. Column chromatography was performed on silica gel (220–400 mesh, Fluka) and silica gel Merck 60 F_254_ plates were used for TLC.

### Scheme A (Figure 10). Examples Demonstrating the General Synthesis of the (10*E*,12*Z*)-Moiety for the Preparation of Compounds 7, 8, 9, 10, and 11

(10*E*,12*Z*)-Octadecadien-1-ol (**7**) was prepared from 10-undecyn-1-ol starting material and coupling with 1-heptyne using the following procedures. (10*E*,12*Z*)-Tetradecadien-1-ol (**8**) was prepared from 10-undecyn-1-ol starting material and coupling with 1-propyne using the following procedures. (8*E*,10*Z*)-Hexadecadien-1-ol (**9**) was prepared from 8-nonyn-1-ol starting material and coupling with 1-heptyne using the following procedures. (5*E*,7*Z*)-Undecadien-1-ol for synthesis of **10** and **11** was prepared using 5-hexyn-1-ol as starting material.

**Figure 10:**
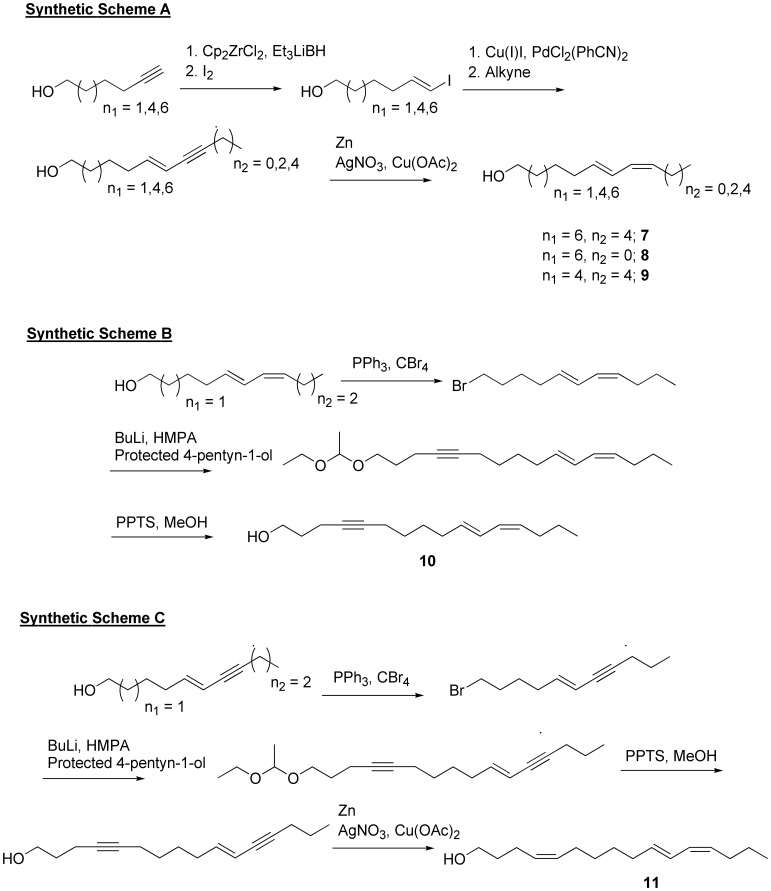
Schemes A–C. Synthetic sequence for preparation of analogues **7**–**11** containing the (*E*,*Z*)-dienyl moiety.

(E)-1-Iodohex-1-en-6-ol. To a solution of Schwartz reagent (8.95 g, 30.6 mmol) in dry THF (40 mL) at room temperature and covered in foil to exclude light, was added super-hydride (30.6 mL, 1 M, 30.6 mmol). The solution was stirred for 1 h after which a lithium salt of 5-hexyn-1-ol, generated from the alcohol (1.5 g, 15.3 mmol) in dry THF (20 mL) at room temperature with super-hydride (15.3 mL, 1 M, 15.3 mmol), was added *via* a canula. After 10 minutes, a solution of iodine (11.7 g, 45.9 mmol) in dry THF (20 mL) was added and the reaction stirred overnight, quenched with sat. NaHCO_3_ solution, extracted into ethyl ether and the combined organic fractions washed with brine, dried (MgSO_4_), filtered and concentrated *in vacuo*. Purification by flash column chromatography (30% ethyl acetate in petroleum ether) afforded a brown oil (3.24 g, 94%). δ_H_ (500 MHz, CDCl_3_) 6.52 (1H, dt, *J* 15.8, 7.1 H-2), 6.01 (1H, d, *J* 15.8, H-1), 3.78 (2H, t, *J* 6.5, H-6), 2.09 (1H, q, *J* 7.0, H-3), 1.60–1.40 (4H, m, H-4,5); δ_c_ (125 MHz, CDCl_3_) 146.3, 74.7, 62.5, 35.8, 31.9, 24.6.

(5*E*)-Undecen-7-yn-1-ol. To a solution of the iodoalkene (3.24 g, 14.4 mmol) and an excess of 1-pentyne (4.9 g, 72.0 mmol) in dry piperidine (60 mL), was added copper (I) iodide (273 mg, 1.44 mmol) and PdCl_2_(PhCN)_2_ (275 mg, 0.72 mmol). The reaction was stirred for 3 days, quenched with aqueous ammonium chloride, extracted into dichloromethane and the combined organic extracts washed with 1M HCl and brine, dried (MgSO_4_) and concentrated *in vacuo*. Purification by flash column chromatography (30% ethyl acetate in petroleum ether) gave a pale brown oil (1.46 g, 61%). δ_H_ (500 MHz, CDCl_3_) 6.04 (1H, dt, *J* 15.8, 7.1 H-5), 5.47 (1H, d, *J* 15.8, H-6), 3.63 (2H, t, *J* 6.5, H-1), 2.26 (1H, t, *J* 6.9, H-9), 2.12 (2H, q, *J* 7.2, H-4), 1.72 (1H, br s, OH), 1.59-1.52 (4H, m, 2CH_2_), 1.29–1.44 (2H, m, CH_2_), 0.99 (3H, t, *J* 7.4, H-11); δ_c_ (125 MHz, CDCl_3_) 147.2, 110.3, 88.8, 79.2, 62.6, 32.6, 32.1, 25.0, 22.3, 21.3, 13.6.

(5*E*,7Z)-Undecadien-1-ol. Fresh zinc dust (8.50 g, 130 mmol) was suspended in water (80 mL) and a solution of Cu(OAc)_2_.2H_2_O (832 mg, 4.58 mmol) in hot water (40 mL) added. After stirring for 15 minutes a solution of AgNO_3_ (961 mg, 5.06 mmol) in hot water (40 mL) was added and the mixture stirred in the dark for 15 minutes. The resulting solid was filtered and washed with water, methanol and diethyl ether before it was dried *in vacuo* and transferred to a solution of (5*E*)-undecen-7-yn-1-ol (800 mg, 4.82 mmol) in water (40 mL) and methanol (60 mL). The reaction was heated at 65°C until starting material was consumed. The reaction was filtered and the filtrate concentrated *in vacuo* before purification by flash column chromatography (20% ethyl acetate in petroleum ether) yielded the diene as a clear, colorless oil (490 mg, 61%). δ_H_ (500 MHz, CDCl_3_) 6.34 (1H, dd, *J* 10.9, 15.0, H-6), 5.98 (1H, t, *J* 10.9, H-7), 5.67 (1H, dt, *J* 7.0, 15.0, H-5), 5.34 (1H, dt, *J* 10.9, 7.6, H-8), 3.67 (2H, t, *J* 6.5, H-1), 2.17-2.14 (4H, m, H-4,H-9), 1.61 (2H, qu, *J* 6.5, H-2), 1.52-1.48 (2H, m, H-3), 1.47-1.39 (2H, m, H-10), 0.93 (3H, t, *J* 7.4, H-11); δ_c_ (125 MHz, CDCl_3_) 134.0, 130.2, 128.6, 126.1, 62.9, 32.6, 32.3, 29.8, 25.5, 22.9, 13.8.

### Scheme B (Figure 10). Synthesis of Ligand 10

(5*E*,7*Z*)-1-Bromoundecadiene. To a solution of (5*E*,7Z)-undecadien-1-ol (450 mg, 2.68 mmol) in dry dichloromethane (20 mL) at 0°C was added triphenylphosphine (0.77 g, 2.94 mmol). Over 5 minutes carbon tetrabromide (0 89 g, 2.68 mmol) was added in portions and the reaction subsequently stirred until complete by TLC. The crude reaction was concentrated *in vacuo* and passed through a short chromatography column eluted with petroleum ether to yield the bromide as a clear, colourless oil (530 mg, 86%). δ_H_ (500 MHz, CDCl_3_) 6.34 (1H, dd, *J* 11.0, 15.1, H-6), 5.98 (1H, t, *J* 11.0, H-7), 5.65 (1H, dt, *J* 7.0, 15.1, H-5), 5.35 (1H, dt, *J* 11.0, 7.6, H-8), 3.44 (2H, t, *J* 6.8, H-1), 2.19-2.14 (4H, m, H-4,H-9), 1.90 (2H, qu, *J* 7.6, H-3), 1.58 (2H, qu, *J* 7.5, H-2), 1.43 (2H, s, *J* 7.4, H-10), 0.95 (3H, t, *J* 7.4, H-11); δ_c_ (125 MHz, CDCl_3_) 133.4, 130.4, 128.5, 126.4, 33.8, 32.3, 31.9, 29.8, 27.9, 22.9, 13.3.

Protected 4-pentyn-1-ol. To a solution of 4-pentyn-1-ol (1.00 g, 11.9 mmol) and ethyl vinyl ether (2 mL) in dichloromethane (20 mL) was added a small spatula of PPTS catalyst. The reaction was stirred overnight, washed with bicarbonate and brine, dried (MgSO_4_), filtered and concentrated *in vacuo* to yield the protected alcohol as a clear colorless oil in quantitative yield. δ_H_ (500 MHz, CDCl_3_) 4.68 (1H, q, *J* 5.3, HCO_2_), 3.68-3.62 (2H, m, CH_2_O), 3.53-3.44 (2H, m, CH_2_O), 2.29 (2H, m, H-3), 1.94 (1H, t, *J* 2.5, H-5), 1.77 (2H, qu, *J* 6.6, H-2), 1.30 (3H, d, *J* 5.4, CH_3_), 1.21 (3H, t, *J* 7.1, CH_3_); δ_c_ (125 MHz, CDCl_3_) 99.6, 83.8, 68.5, 63.2, 60.8, 28.7, 19.8, 15.3, 15.3.

Protected (10*E*,12Z)-Hexadecadien-4-yn-1-ol. The alkyne (200 mg, 1.28 mmol) was dissolved in dry THF (5 mL) and HMPA (1 mL) at −50°C under nitrogen. n-Butyllithium (0.60 mL, 2.5 M, 1.50 mmol) was added and the reaction stirred for 45 minutes before the addition of the bromide (295 mg, 1.28 mmol) in dry THF (1 mL) after which the reaction was stirred overnight while warming to room temperature. The reaction was diluted with water and extracted with Et_2_O and the organic fractions combined, dried (MgSO_4_), concentrated *in vacuo* and purified by flash column chromatography (5% ethyl acetate in petroleum ether) to yield a clear colorless oil (95 mg, 25%). δ_H_ (500 MHz, CDCl_3_) 6.30 (1H, dd, *J* 11.0, 15.1, H-11), 5.96 (1H, t, *J* 11.0, H-12), 5.65 (1H, dt, *J* 7.0, 15.1, H-10), 5.32 (1H, dt, *J* 11.0, 7.6, H-13), 4.70 (1H, q, *J* 5.3, CHO_2_), 3.69-3.64 (2H, m, CH_2_O), 3.54-3.47 (2H, m, CH_2_O), 2.29-2.25 (2H, m, CH_2_), 2.17-2.11 (6H, m, 3CH_2_), 1.75 (2H, qu, *J* 6.6, CH_2_), 1.51-1.48 (4H, m, 2CH_2_), 1.41 (2H, s, *J* 6.6, CH_2_), 1.32 (3H, d, *J* 5.3, CH_3_), 1.22 (3H, t, *J* 7.1, CH_3_), 0.91 (3H, t, *J* 7.4, CH_3_); δ_c_ (125 MHz, CDCl_3_) 134.1, 130.0, 128.7, 125.9, 99.6, 80.4, 79.5, 63.6, 60.7, 32.4, 29.8, 29.3, 28.6, 28.6, 22.9, 19.8, 18.6, 15.6, 15.3, 13.8.

(10*E*,12Z)-Hexadecadien-4-yn-1-ol (**10**). Deprotection was performed using a small spatula of PPTS catalyst in a methanolic (15 mL) solution of the substrate (35 mg, 0.11 mmol) overnight. Flash column chromatography (20% ethyl acetate in petroleum ether) yielded **10** as a clear, colorless oil (25 mg, 97%). δ_H_ (500 MHz, CDCl_3_) 6.33 (1H, dd, *J* 11.0, 15.1, H-11), 5.98 (1H, t, *J* 11.0, H-12), 5.66 (1H, dt, *J* 7.0, 15.1, H-10), 5.33 (1H, dt, *J* 11.0, 7.6, H-13), 3.78 (2H, t, *J* 6.1, H-1), 2.32-2.28 (2H, m, CH_2_), 2.18-2.11 (6H, m, 3CH_2_), 1.76 (2H, qu, *J* 6.5, CH_2_), 1.52-1.48 (4H, m, 2CH_2_), 1.41 (2H, sextet, *J* 6.6, CH_2_), 0.94 (3H, t, *J* 7.4, CH_3_); δ_c_ (125 MHz, CDCl_3_) 134.1, 130.0, 128.7, 126.0, 80.9, 79.5, 62.1, 32.4, 31.6, 29.8, 28.6, 28.6, 22.9, 18.6, 15.5, 13.8.

### Scheme C (Figure 10). Synthesis of Ligand 11

(10*E*)-Hexadecen-4,12-diyn-1-ol. The alkyne (234 mg, 1.50 mmol) was dissolved in dry THF (3 mL) and HMPA (1 mL) at −50°C under nitrogen. n-Butyllithium (0.72 mL, 2.5 M, 1.8 mmol) was added and the reaction stirred for 45 minutes before the addition of the bromide (270 mg, 1.18 mmol) in dry THF (1 mL) after which the reaction was stirred overnight while warming to room temperature. The reaction was diluted with water and extracted with Et_2_O and the organic fractions combined, dried (MgSO_4_), concentrated *in vacuo* and purified by flash column chromatography (5% ethyl acetate in petroleum ether) to yield the protected enediyne. The material was immediately treated with a methanolic solution of *p*-toluenesulfonic acid catalyst for I h. After dilution with water, the product was extracted with Et_2_O and purified by flash column chromatography (20% ethyl acetate in petroleum ether) to yield the enediyne as a clear colorless oil (116 mg, 42% over 2 steps). δ_H_ (500 MHz, CDCl_3_) 6.03 (1H, dt, *J* 15.8, 7.1, H-10), 5.46 (1H, d, *J* 15.8, H-11), 3.73 (2H, t, *J* 6.1, H-1), 2.28-2.24 (4H, m, 2CH_2_), 2.14-2.08 (4H, m, 2CH_2_), 1.99 (1H, br s, OH), 1.72 (2H, qu, *J* 6.6, CH_2_), 1.52 (2H, sextet, *J* 7.3, CH_2_), 1.48-1.46 (4H, m, 2CH_2_), 0.98 (3H, t, *J* 7.4, CH_3_); δ_c_ (125 MHz, CDCl_3_) 142.8, 110.1, 88.7, 80.6, 79.6, 79.2, 61.9, 32.4, 31.6, 28.4, 28.0, 22.3, 21.3, 18.5, 15.4, 13.6.

(4*Z*,10*E*,12*Z*)-Hexadecatrien-1-ol (**11**). Fresh zinc dust (1.76 mg, 27.0 mmol) was suspended in water (15 mL) and a solution of Cu(OAc)_2_.2H_2_O (173 mg, 0.95 mmol) in hot water (8 mL) added. After stirring for 15 minutes a solution of AgNO_3_ (199 mg, 1.05 mmol) in hot water (8 mL) was added and the mixture stirred in the dark for 15 minutes. The resulting solid was filtered and washed with water, methanol and diethyl ether before it was dried *in vacuo* and transferred to a solution of (10*E*)-hexadecen-4,12-diyn-1-ol (116 mg, 0.50 mmol) in water (10 mL) and methanol (15 mL). The reaction was heated at 65°C until starting material was consumed. The reaction was filtered and the filtrate concentrated *in vacuo* before purification by flash column chromatography (20% ethyl acetate in petroleum ether) yielded the triene (**11**) as a clear, colorless oil (9 mg, 8%). The major product of the reaction was compound **10** from incomplete reduction. δ_H_ (500 MHz, CDCl_3_) 6.33 (1H, dd, *J* 11.0, 15.2, H-11), 5.98 (1H, t, *J* 11.0, H-12), 5.67 (1H, dt, *J* 15.1, 7.0, H-10), 5.45-5.38 (2H, m, H-4, H-5), 5.34 (1H, dt, *J* 15.2, 7.6, H-13), 3.68 (2H, t, *J* 6.5, H-1), 2.19-2.2.09 (6H, m, H-3, H-8, H-14), 2.06 (2H, q, *J* 6.2, H-6), 1.66 (2H, qu, *J* 7.1, H-2), 1.47-1.35 (4H, m, H-7, H-15), 0.94 (3H, t, *J* 7.4, CH_3_); δ_c_ (125 MHz, CDCl_3_) 134.4, 130.6, 130.0, 129.0, 128.7, 125.8, 62.7, 32.8, 32.7, 29.8, 29.3, 29.0, 27.1, 23.6, 22.9, 13.8.

### Scheme D (Figure 11). General Synthesis of the Diyne-moiety for the Preparation of Compound 6

1-Iodopent-1-yne. 1-Pentyne (1.00 g, 14.7 mmol) was dissolved in dry Et_2_O (15 mL) at −78°C under nitrogen. n-Butyllithium (5.87 mL, 2.5 M, 14.68 mmol) was added drop wise and the reaction stirred for 1 h. Iodine (4.10 g, 16.2 mmol) was added in dry Et_2_O and the mixture was stirred overnight. The reaction was quenched (aqueous ammonium chloride), extracted into Et_2_O, and the combined organic extracts washed with sodium thiosulfate, brine, dried (MgSO_4_) and concentrated *in vacuo* to yield the crude product as a clear colorless oil (2.70 g, 95%). δ_H_ (500 MHz, CDCl_3_) 2.36 (2H, t, *J* 7.1, H-3), 1.56 (2H, s, *J* 7.2, H-4), 1.00 (3H, t, *J* 7.4, H-5); δ_c_ (125 MHz, CDCl_3_) 94.7, 22.8, 22.0, 13.5, -7.3.

**Figure 11:**
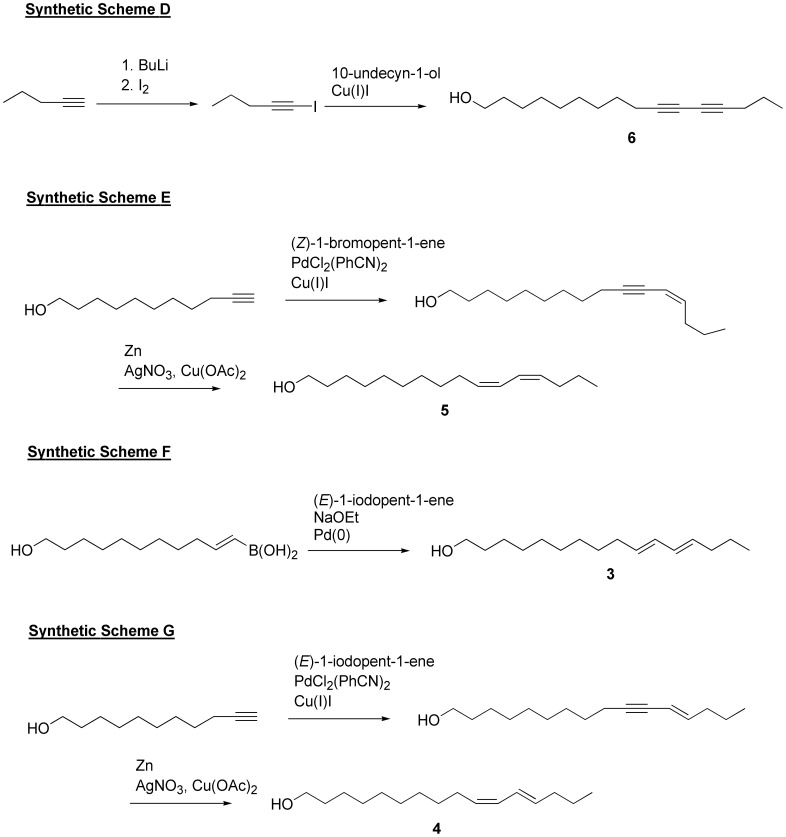
Schemes D–G. Synthetic sequence for preparation of analogues **3**–**6** differing in unsaturation.

10,12-Hexadecadiyn-1-ol. Without further purification 1-iodopent-1-yne (300 mg, 1.55 mmol) and 10-undecyn-1-ol (200 mg, 1.19 mmol) were dissolved in dry pyrrolidine (5 mL) at 0°C. Copper (I) iodide (230 mg, 1.19 mmol) was added and the mixture stirred overnight before separation between water and dichloromethane. The combined organic fractions were washed with brine and concentrated *in vacuo* before purification by flash column chromatography (25% ethyl acetate in petroleum ether) to yield the product (**6**) as a white, waxy solid (276 mg, 99%); δ_H_ (500 MHz, CDCl_3_) 3.64 (2H, t, *J* 6.7, H-1), 2.26-2.22 (4H, m, H-9, H-11), 1.58-1.50 (6H, m, 3CH_2_), 1.39-1.25 (10H, m, 5CH_2_), 1.00 (3H, *t*, J 7.4, H-16); δ_c_ (125 MHz, CDCl_3_) 77.7, 77.6, 65.4, 65.2, 63.0, 32.8, 29.4, 29.4, 29.0, 28.8, 28.3, 25.7, 21.2, 21.1, 19.2, 13.5.

### Scheme E (Figure 11). Synthesis of (10*Z*,12*Z*)-hexadecadien-1-ol (5)

(12*Z*)-Hexadecen-10-yn-1-ol. To a solution of 10-undecyn-1-ol (271 mg, 1.61 mmol) in piperidine (7 mL) was added 1-bromopent-1-ene (240 mg, 1.61 mmol). Copper (I) iodide (31 mg, 0.16 mmol) and PdCl_2_(PhCN)_2_ (31 mg, 0.08 mmol) were added and the mixture was stirred until the starting material was consumed, then quenched with aqueous ammonium chloride and extracted with dichloromethane. The combined organic extracts were washed with 1 M HCl and brine, dried (MgSO_4_), filtered and concentrated *in vacuo*. Purification by flash column chromatography (15% ethyl acetate in petroleum ether) yielded the enyne as a clear, colorless oil (336 mg, 88%).

(10*Z*,12*Z*)-hexadecadien-1-ol (**5**). The enyne, (12*Z*)-hexadecen-10-yn-1-ol (290 mg, 1.23 mmol), was reduced using the Zn/Ag/Cu amalgam described above to yield **5** as a clear, colorless oil (240 mg, 89%). δ_H_ (500 MHz, CDCl_3_) 6.28-6.21 (2H, m, H-11, H-12), 5.46-5.41 (2H, m, H-10, H13), 3.62 (2H, t, *J* 6.5, H-1), 2.17-2.01 (4H, m, H-9, H-14), 1.55 (2H, qu, *J* 7.3, CH_2_), 1.42-1.23 (14H, m, 7CH_2_), 0.89 (3H, *t*, *J* 7.4, H-16).

### Scheme F (Figure 11). Synthesis of (10*E*,12*E*)-hexadecadien-1-ol (3)

(10*E*,12*E*)-hexadecadien-1-ol (**3**). To a solution of the boron compound (400 mg, 1.96 mmol) and Pd(Ph)_3_ (107 mg, 0.09 mmol) in benzene was added vinyl iodide (400 mg, 2.04 mmol) and sodium ethoxide in ethanol (21% in 2 mL). The mixture was refluxed for 2 hours, then pre-absorbed onto silica gel for flash column chromatography (10% ethyl acetate in petroleum ether) to yield the diene as a clear, colorless oil (240 mg, 54%). δ_H_ (500 MHz, CDCl_3_) 6.02-5.96 (2H, m, H-11, H-12), 5.58-5.52 (2H, m, H-10, H-13), 3.62 (2H, t, *J* 6.5, H-1), 2.05-2.00 (4H, m, H-9, H-14), 1.55 (2H, qu, *J* 6.7, CH_2_), 1.41-1.26 (14H, m, 7CH_2_), 0.87 (3H, t, *J* 7.4, CH_3_); δ_c_ (125 MHz, CDCl_3_) 132.4, 132.2, 130.5, 130.3, 63.1, 34.7, 32.8, 32.6, 29.5, 29.5, 29.4, 29.4, 29.2, 25.7, 22.6, 13.7.

### Scheme G (Figure 11). Synthesis of (10*Z*,12*E*)-hexadecadien-1-ol (4)

(12*E*)-hexadecen-10-yn-1-ol. To a suspension of vinyl iodide (1.16 g, 5.90 mmol), Cu(I) iodide (113 mg, 0.59 mmol) and PdCl_2_(PhCN)_2_ (114 mg, 0.30 mmol) in piperidine (20 mL) was added 10-undecyn-1-ol (1.00 g, 5.9 mmol). The reaction was stirred for 5 h until the starting material was consumed. After dilution with water and extraction with dichloromethane, the organic fraction was washed with 1 M HCl and brine, dried (MgSO_4_) and concentrated *in vacuo*. Flash column chromatography (10% ethyl acetate in petroleum ether) yielded the product as a clear, colorless oil (1.16 g, 83%).

(10*Z*,12*E*)-hexadecadien-1-ol (**4**). The enyne, (12*E*)-hexadecen-10-yn-1-ol (935 mg, 3.95 mmol), was reduced using the Zn/Ag/Cu amalgam described above to yield **4** as a clear, colorless oil (895 mg, 95%). δ_H_ (500 MHz, CDCl_3_) 6.28 (1H, dd, *J* 11.0, 15.0, H-12), 5.93 (1H, t, *J* 11.0, H-11), 5.64 (1H, dt, *J* 7.0, 15.0, H-13), 5.29 (1H, dt, *J* 11.0, 7.6, H-10), 3.61 (2H, t, *J* 6.7, H-1), 2.13 (2H, q, *J* 6.8, CH_2_), 2.06 (2H, q, *J* 7.2, CH_2_), 1.54 (2H, qu, *J* 6.9, CH_2_), 1.48 (1H, br s, OH), 1.44-1.24 (14H, m, 7CH_2_), 0.89 (3H, t, *J* 7.4, CH_3_); δ_c_ (125 MHz, CDCl_3_) 134.4, 130.1, 128.5, 125.7, 63.0, 34.9, 32.7, 29.7, 29.5, 29.4, 29.4, 29.2, 27.6, 25.7, 22.5, 13.7.

### Receptor Cloning

Full-length BmorOR1 and BmorOrco gene sequences were amplified from constructs available from previous works in our laboratory [18], [19]. They were transferred into pBlueScript by standard procedures and then subcloned into pGEMHE [20], and their sequences were confirmed by DNA sequencing (Davis Sequencing Center, Davis, CA).

### In vitro Transcription Oocyte and Microinjection

In vitro transcription of cRNAs (BmorOR1 and BmorOrco) was performed by using a mMESSAGE mMACHINE T7 Kit (Ambion) according to the manufacturer’s protocol. Plasmids were linearized with Nhe I, and capped cRNA was transcribed using T7 RNA polymerase. The cRNAs were purified with LiCl precipitation solution and re-suspended in nuclease-free water at a concentration of 200 ug/ml and stored at −80°C in aliquots. RNA concentrations were determined by UV spectrophotometry. cRNA were microinjected (2 ng of a receptor cRNA and 2 ng of an Orco cRNA) into *Xenopus laevis* oocytes on stage V or VI (EcoCyte Bioscience, Austin TX). The oocytes were then incubated at 18°C for 3–7 days in modified Barth’s solution [in mM: 88 NaCl, 1 KCl, 2.4 NaHCO_3_, 0.82 MgSO_4_, 0.33 Ca(NO_3_)_2_, 0.41 CaCl_2_, 10 HEPES, pH 7.4] supplemented with 10 µg/ml of gentamycin, 10 µg/ml of streptomycin and 1.8 mM sodium pyruvate.

### Protein Expression and Purification

BmorPBP1 was prepared and purified, as previously described [14]. Lyophilized protein was dissolved in 1X Ringer’s solution (see below) to make 2 mg/ml samples.

### Sample Preparations and Electrophysiological Recordings

Stock solutions were prepared in dimethyl sulfoxide (DMSO) and stored at −20°C, if they could not be used immediately. An aliquot of each solution was taken, diluted with hexane, and analyzed by gas chromatography-mass spectrometry using analytical instrumentation, column and conditions previously described [14]. The oven was operated at 70°C, held at this initial temperature for 1 min, increased to 290°C at 10°C/min, and held at this final temperature for 10 min. Prior to electrophysiological measurements, stock solutions were brought to room temperature and diluted in 1X Ringer’s solution [in mM: NaCl 96, KCl 2, CaCl_2_ 1.8, MgCl_2_ 1, HEPES 5, pH 7.6] containing 0.1% DMSO, except for preparation with BmorPBP1, which were diluted with the same buffer without DMSO. Two equal aliquots from the same initial solution (either bombykol or bombykal) were transferred to different vials from which decadic dilutions were made. One of the samples was diluted with Ringer-DMSO and the other was similarly diluted with Ringer-PBP. Thus, comparisons were made with samples derived from the same mother solution at the same concentration, but differing only in the solubilizer (solvent vs. PBP). All ligand solutions were freshly prepared and discharged if not used within 20 min. Chemical-induced currents were recorded with the two-electrode voltage-clamp technique at holding potential of −80mV. Signals were amplified with an OC-725C amplifier (Warner Instruments, Hamden, CT), low-pass filtered at 50 Hz and digitized at 1 kHz. Data acquisition and analysis were carried out with Digidata 1440A and software pCLAMP 10 (Molecular Devices, LLC, Sunnyvale, CA).
